# Two Approaches to Enhance the Processivity and Salt Tolerance of *Staphylococcus aureus* DNA Polymerase

**DOI:** 10.1007/s10930-019-09818-7

**Published:** 2019-02-13

**Authors:** Bing Zhai, Joseph Chow, Qi Cheng

**Affiliations:** Biotechnology Research Institute, Chinese Academy of Agricultural Sciences, Beijing, 100081 China

**Keywords:** Processivity, Salt tolerance, Recombinase polymerase amplification, Thioredoxin-binding domain, Sso7d

## Abstract

In this article, two engineering-strategies were carried out to enhance the processivity of the DNA polymerase used in recombinase polymerase amplification (RPA). We demonstrate that covalent linkage of a non-specific, double-stranded DNA binding protein, Sso7d, to the large fragment of *Staphylococcus aureus* Pol I (Sau) caused a moderate enhancement of processivity and a significant improvement in the salt tolerance of Sau. Meanwhile, we provide evidence suggesting that insertion of the thioredoxin-binding domain from bacteriophage T7 DNA polymerase into the analogous position of the large fragment of Sau dramatically enhanced the processivity and mildly increased the salt tolerance of Sau when a host DNA binding protein, thioredoxin, was annexed. Both of these two strategies did not improve the amplifying performance of Sau in RPA, indicating that optimum processivity is crucial for amplifying efficiency.

**Electronic supplementary material:**

The online version of this article (10.1007/s10930-019-09818-7) contains supplementary material, which is available to authorized users.

## Introduction

In vivo, DNA polymerases play key roles in replicating the genome and repairing DNA to preserve the genetic information in all species. Processivity is a crucial character for DNA polymerase, as it defines the average number of nucleotides added by a polymerase enzyme per association event with the template strand. DNA polymerases in charge of replication tasks are very processive, frequently carrying out multiple catalytic cycles before dissociating from the template DNA [1]. While non-replicative polymerases, such as polymerases that fill in gaps and repair damage, are usually not very processive [2].

Most polymerases rely on specific accessory proteins to achieve high processivity. Eukaryotic replicative polymerases, *Escherichia coli* (*E. coli*) Pol III and bacteriophage T4 DNA polymerase are bound by the ring- shaped multimeric “DNA sliding clamp” to encircle the DNA [3–6]. The DNA binding protein UL42 is essential for the maintenance of the processivity of herpes simplex virus (HSV) DNA polymerase [7, 8]. Bacteriophage T7 DNA polymerase forms a stable 1:1 complex with thioredoxin from *E. coli*, which helps to increase the processivity of the polymerase from less than 15 nt to more than 2000 nt [9].

Over the past decades, DNA polymerases have been extensively studied due to their wide applications in in vitro DNA amplification. The thermostable Taq polymerase from *Thermus aquaticus* is the first and most widely used DNA polymerase in the polymerase chain reaction (PCR); therefore, the functional engineering of DNA polymerases are mainly focused on Taq [10]. Different engineering strategies to create fusion proteins have been undertaken, and Taq variants with enhanced processivity or improved PCR performance were generated [11–14]. Nevertheless, functional engineering studies on hypothermal polymerases are rare.

Recombinase polymerase amplification (RPA) is an isothermal nucleic amplification technique that was first established by Piepenburg et al. RPA involves exponential amplification with no pre-treatment of sample DNA that could be achieved at constant low temperature in a relatively short time [15]. When compared with the other existing isothermal amplification techniques, RPA is exemplary because it does not require complex primer-designing skills [16] or sophisticated enzymatic steps [17] and does not have strict limitations for the length of products [18, 19]. Therefore, RPA can be a competitive DNA amplification technique, especially for DNA detection and amplification in the field of points of care. The polymerase optimized in RPA is the large fragment of *Bacillus subtilis* Pol I (Bsu) or *Staphylococcus aureus* Pol I (Sau) [15, 20], which are non-replicative polymerases with low processivity, the size of amplicons of RPA is limited within 1000 bp. Enhancing the processivity of these polymerases might improve the amplifying performance of RPA and benefit the use of RPA in a broader area.

In the present study, we used structural alignment to design two Sau fusions and PCR amplification to construct them. Fusions were inserted into selective vectors, and expressed in certain strains of *E. coli*. to increase solubility. To verify the performance of the fusion proteins, they were assayed for overall activity, processivity, salt tolerance, and amplifying efficiency.

## Materials and Methods

### General Reagents and Equipment

Restriction enzymes were purchased from New England Biolabs. T4 DNA ligase and PrimeSTAR HS DNA polymerase were purchased from Takara. Oligonucleotides were synthetized by BGI, China. DNA Engine Thermal Cyclers (MJ Research) were used for PCR and an AKTA purifier (Amersham Pharmacia Biotech) was used for protein purification.

### Bacterial Strains and Plasmids

*Escherichia coli* strains DH5α, BL21 (DE3), M15 and plasmids containing the *UvsX, UvsY, gp32* and *Sau* genes were obtained from the laboratory collection.

### Construction of Sso7d-Sau

*Sso7d* with a flexible linker at its 3′ side was synthesized and conjugated to *Sau* at its 5′ side. Oligonucleotides Sso_F and Linker_R (Table 1) were used to amplify the *Sso7d* with the linker and oligonucleotides PS_F and PS_R (Table 1) were used to amplify *Sau* from the plasmid containing the *Sau* gene. Overlap PCR was carried out with the oligonucleotides Sso_F and PS_R to obtain the entire recombinant sequence with a His-tag at the 5′ side and two restriction sites, NcoI and BamHI, at the respective ends. The entire recombinant sequence was cloned into the pTrc99A vector, and the recombinant protein was designated Sso7d-Sau.

**Table 1:** Oligonucleotides used

Oligo name	Oligo sequence
Construction of *Sso7d-Sau*	
Sso_F	5′-CATGCCATGGCAACAGTAAAGTTCAA-3′
Linker_R	5′-GTCGACGCTTGCTGATGAGCCTCCG-3′
PS_F	5′-CGGAGGCTCATCAGCAAGCGTTG-3′
PS_R	5′-TTTTCCTTTTGCGGCCGCTTTTGCATCATACCAAGTTGC-3′
Generation of hybrid *Sau-TBD*	
PT1_F	5′-CATGCCATGGAACATCATCATCATCATCATTCAGCAAGCGTTGAAG-3′
PT1_R	5′-GATACCACGA ACCAGCTGCATCATGGAT-3′
PT2_F	5′-GTTGTGTTTAACCCTTCGTCTCCTAAGCAATTAGGTG − 3′
PT2_R	5′-CGCGGATCCTTATTTTGCATCATACC-3′
T BD_F	5′-TGCAGCTGGTTCGTGGTATCAGCCTAAAGG − 3′
TBD_R	5′-CACCTAATTGCTTAGGAGACGAAGGGTTAAACACAAC-3′
Amplification of *TrxA*	
Trx_F	5′-CATGCCATGGGCAGCGATAAAATTATTCACCTG-3′
Trx_R	5′-TTTTCCTTTTGCGGCCGCCGCCAGGTTAGCGTCGAGG-3′
Primer used for polymerase activity assay	
M13-40	5′-GTTTTCCCAGTCACGACG-3′
Primer used for processivity assay	
M13-40 LF	5′-FAM-GTTTTCCCAGTCACGACGTTGTAAAACGACGGCC-3′
Amplifying efficiency assay	
200_F	5′-AATTTGCTGAGATTAACATAGTAGTCAATG-3′
200_R	5′-ACAATGTTTTATCTTACTGTCTTTGATGAG-3′
500_F	5′-ACTACTAAATCCTGAATAGCTTTAAGAAGG-3′
500_R	5′-CAGAAAGCTAAATATGGAAAACTACAATAC-3′
600_F	5′-TGAGTATTGGTTTATTTGGCGATTATTATC-3′
600_R	5′-AAATAATTCCTGAAGATATTAAAGAGCGTC-3′
700_F	5′-CTCAAAAGGTATAGTTAAATCACTGAATCC-3′
700_R	5′-AGAAAGCTAAATATGGAAAACTACAATACG-3′
800_F	5′-TTTTGAATAATAAATGTTACTGTTCTTGCG-3′
800_R	5′-AATTATTGGAAAAGAGTTATGTATCAGTGC-3′
900_F	5′-TGAGTATTGGTTTATTTGGCGATTATTATC-3′
900_R	5′-TGCACAAAAGAAATTACCTTCATATTTAAC-3′
1000_F	5′-CCCATCGTCTTTCTGATTTAATAATAGATG-3′
1000_R	5′-CAGAGGGATCTAGAATATGATGAAAGATAG-3′

### Generation of the Hybrid Sau-TBD

A homology modelling method was employed to predict the protein structure of T7 DNA polymerase and Sau. The crystal structure of T7 DNA polymerase (PDB: 2AJQ_A) and a structural homology of Sau (PDB: 4DQQ_D) were generated using Swiss-model (http://swissmodel.expasy.org/). Comparisons of the 3D structures of T7 DNA polymerase and the structural homology of Sau were performed using DaliLite from EBI (http://www.ebi.ac.uk/Tools/structure/dalilite/). The amino acids 237–242 of Sau were accordingly replaced by the synthesized TBD of T7 DNA polymerase through overlap PCR. Oligonucleotides PT1_F and PT1_R were used to amplify the first portion of *Sau*, PT2_F and PT2_R were used to amplify the second portion of *Sau*, and TBD_F and TBD_R were used to amplify the TBD sequence. PT1_F and TBD_R were used to accomplish the overlap between the first fragment of *Sau* and the TBD sequence fragment; and PT1_F and PT2_R were used to amplify the entire hybrid sequence with a His-tag at the 5′ side and two restriction sites, NcoI and BamHI, at the ends. The oligonucleotides used are listed in Table 1. The entire hybrid sequence was cloned into the pTrc99A vector, and the hybrid protein was designated Sau-TBD.

### Construction of TrxA-pET28a

*TrxA* with restriction sites NcoI and NotI at the ends was amplified using the oligonucleotides Trx_F and Trx_R (Table 1) and the genomic DNA of *E. coli* and cloned into the pET28a vector.

### Expression and Purification of Proteins

Plasmid DNAs containing *Sso7d-Sau, Sau-TBD* and *TrxA* were transformed into *E. coli* strain BL21 (DE3), and those containing *UvsX, UvsY, gp32* and *Sau* were transformed into *E. coli* strain M15.

The transformed bacteria were respectively grown in 500 ml of TB medium in the presence of the corresponding antibiotics until the OD_600_ reached 0.3. IPTG was added to 1 mM to induce expression at 37 °C for 4 h. Cleared lysate was prepared through ultrasonication in Ni-NTA binding buffer (20 mM sodium phosphate, 0.5 M NaCl, 10 mM imidazole, pH 7.4), followed by centrifugation at 12,000 rpm for 30 min at 4 °C. The supernatant was loaded onto the pre-equilibrated Ni Sepharose 6 Fast Flow column (1 ml, GE Healthcare), and the bound proteins were step-eluted using elution buffer (20 mM sodium phosphate, 0.5 M NaCl, 500 mM imidazole, pH 7.4). The fractions were then pooled and purified on a HiTrap Heparin HP column (5 ml, GE Healthcare) with binding buffer (10 mM sodium phosphate, pH 7.0) and elution buffer (10 mM sodium phosphate, 1 M NaCl, pH 7.0). The peak fractions contained a single polypeptide, and the apparent molecular weight was verified using SDS-PAGE with Coomassie blue staining and molecular weight markers (Pierce Unstained Protein Molecular Weight Marker, Thermo). The peak fractions of Sau, Sso7d-Sau, Sau-TBD and thioredoxin were dialyzed against pre-storage buffer P (10 mM Tris-HCl pH 7.5, 50 mM KCl, 1 mM DTT, 1 mM EDTA, 0.1% NP-40), and UvsX, UvsY and gp32 were dialyzed against pre-storage buffer X (20 mM Tris- HCl pH 7.5, 500 mM NaCl, 0.5 mM DTT, 1 mM EDTA).

### Polymerase Activity Assay

For the test reaction of Sau, Sso7d-Sau and Sau-TBD, oligonucleotide M13-40 (Table 1) was pre-annealed to the ssM13mp18 DNA template (New England Biolabs Inc.) in 2 × standard RPA reaction solution, and 300 µM dNTPs and 1.5 nM polymerase were added to the mixture after the annealing. Thioredoxin was added to a final concentration of 1.5 µM when indicated. Other reagents were used according to a standard RPA reaction described in the US patent NO 2012/0129173 A1 [21], and 14 mM MgAc was added last to initiate the reaction at 37 °C. For the control reaction, M13-40 was pre-annealed to the ssM13mp18 DNA template in 10 × ThermoPol Buffer (New England Biolabs Inc.). The *Bst* DNA polymerase large fragment (Bst, New England Biolabs Inc.) was used at 1.5 nM, and dNTPs were added to a final concentration of 300 µM. The control reaction was carried out at 65 °C. Samples were obtained at different time points and added to an equal volume of 1:200 dilution of PicoGreen (Invitrogen) in TE (10 mM Tris-HCl pH 8.0 and 1.0 mM EDTA pH 8.0). The amount of DNA that was synthesized in each reaction was quantified using a fluorescence plate reader (Fluostar Optima, BMG LABTECH). The unit activity of Sau, Sso7d-Sau and Sau-TBD (−/+ thioredoxin) were determined by comparing their initial rates with that of Bst.

### Processivity Assay

A 5′ FAM-labelled primer, M13-40LF (Table 1), was pre-annealed to ssM13mp18 DNA in 2 × standard RPA reaction solutions and then mixed with 300 mM dNTPs. Different molar ratios, from 1:100 to 1:2000, of primed template to DNA polymerase were used to reach the processivity condition; usually, a lower molar ratio is requested for processive enzymes than for non-processive enzymes. Thioredoxin was added to a final concentration of 50 μM when indicated. Other reagents were used according to a standard RPA reaction described in the US patent NO 2012/0129173 A1 [21] and 14 mM MgAc was added last into a 20 μl total volume at last to initiate the reaction at 37 °C. Samples were obtained at different time points to avoid multiple binding and extension of the same primer, diluted in gel loading dye and analyzed on a DNA sequencer (3730 × l DNA Analyzer, Applied Biosystems). The median length of the products of each reaction was determined following the method described by Wang et al. [14]. The probability of the polymerase not terminating at a specific position of the template (PI) and the average extension length were determined using the analysis method described by Von Hippel et al. [22].

### Amplifying Efficiency Assay

Lambda phage DNA at 130 pg/μl, used as the template, and five pairs of primers were used to amplify different sized amplicons (Table 1) to assess the amplifying efficiency of the original and modified DNA polymerases in RPA. In the control reactions, the reaction mixture was prepared according to US patent NO 2012/0129173 A1 [21]. In the test reactions, Sso7d-Sau at 33.4 ng/μl or Sau-TBD at 33.2 ng/μl was used instead of Sau to maintain a consistent molar ratio. The optimum concentration of thioredoxin to stimulate Sau-TBD in the standard RPA reaction was determined (Fig. S1), and thioredoxin was used at a concentration of 50 μM when indicated. Other reagents were used according to a standard RPA reaction. Reactions were carried out at 37 °C for 40 or 80 min, as indicated. After the reaction was accomplished, an equal volume of phenol chloroform was added to extract DNA, and 5 μl of the supernatant was loaded onto a 1.2% agarose gel.

### Salt Tolerance Assay

Lambda phage DNA at 130 pg/μl was used as template, and oligonucleotides 500_F and 500_R (Table 1) were used as primer pairs. In the control reaction, the reaction mixture was prepared according to US patent NO 2012/0129173 A1 [21]. In the test reactions, Sso7d-Sau at 33.4 ng/μl or Sau-TBD at 33.2 ng/μl was used instead of Sau to maintain a consistent molar ratio, and thioredoxin was used at a concentration of 50 μM when indicated. Different concentrations of KAc, as indicated, were added to each reaction to analyze the salt tolerance ability of Sau, Sso7d-Sau and Sau-TBD (−/+ thioredoxin). The reactions were carried out at 37 °C for 40 min. After each reaction was accomplished, the samples were mixed with an equal volume of phenol chloroform to allow extract DNA, and 5 μl of the supernatant was loaded onto a 1.2% agarose gel.

## Results

### Strategy to Enhance the Processivity of the Sau DNA Polymerase

**Fig. 1:**
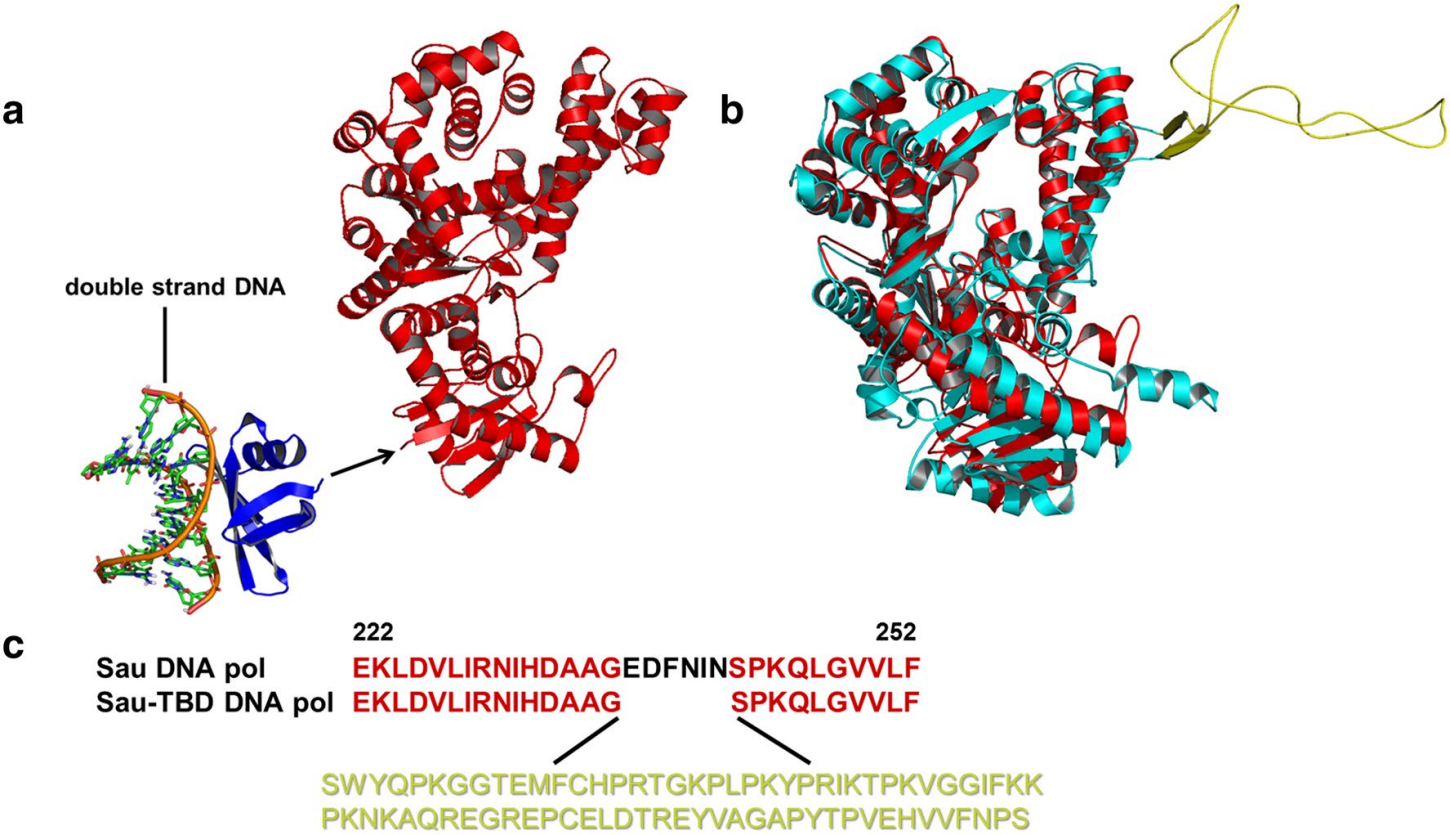
**a** Three-dimensional structure of Sso7d (blue) and the homology of Sau (red). The arrow indicates where Sso7d was linked to Sau. The dsDNA where Sso7d binds is shown in multiple colors. **b** Superimposition of the three-dimensional structure of T7 DNA polymerase (cyan) with the structural homology of Sau (red). The thioredoxin-binding domain of T7 DNA polymerase is highlighted in yellow. **c** The amino acid sequence of Sau from residue 222 to 252, is indicated in red, and the sequence of the thioredoxin-binding domain of T7 DNA polymerase (yellow) was inserted into the region where 6 amino acids were deleted (black). (Color figure online)

A non-specific, double-stranded DNA binding protein, Sso7d, which was proven to be efficient in enhancing the processivity of DNA polymerases from families A and B [14], was fused to the N terminus of Sau (Fig. 1a). It was proved that hypothermal DNA binding protein could successfully work in a hyperthermal environment [11], therefore, it was expected that, the hyperthermal protein Sso7d would at least partially perform DNA-binding function at lower temperatures.

Meanwhile, 3D structure of the replicative T7 DNA polymerase [9] and a structural homolog of Sau were generated and compared (Fig. 1b). Seventy-six residues of the T7 DNA polymerase TBD were accordingly inserted into Sau in the analogous position (Fig. 1c). The new hybrid enzyme was expected to achieve high processivity when stimulated by the DNA binding accessory protein thioredoxin. All of the proteins used in this article were purified to homogeneity using immobilized metal affinity followed by heparin chromatography.

### Comparison of DNA Polymerase Activity Among Sau, Sso7d-Sau and Sau-TBD (−/+ Thioredoxin)

The DNA polymerase activity was compared between the wild type Sau and engineered Sau variants using primed M13mp18 DNA (Table 2). The fusion protein Sso7d-Sau only displayed approximately 23% activity compared with the wild type Sau.

**Table 2:** Results of the DNA polymerase activity assay

Polymerase	Polymerase activity (units/mg protein)	
	− Thioredoxin	+ Thioredoxin
Sau	3089	3099
Sso7d-Sau	719	
Sau-TBD	1316	2864

One unit of polymerase activity corresponds to the incorporation of 10 nmoles of all four nucleotides within 30 min at 37 °C

In the absence of thioredoxin, the Sau-TBD hybrid protein displayed approximately 43% activity compared with the wild type Sau, and when stimulated by thioredoxin, the activity of the Sau-TBD hybrid protein was enhanced more than twofold, while there was no significant activity enhancement for Sau. In the presence of thioredoxin, the Sau-TBD hybrid protein exhibited approximately 93% activity compared with the wild type Sau.

### Comparison of Processivity Among Sau, Sso7d-Sau and Sau-TBD (−/+ Thioredoxin)

**Fig. 2:**
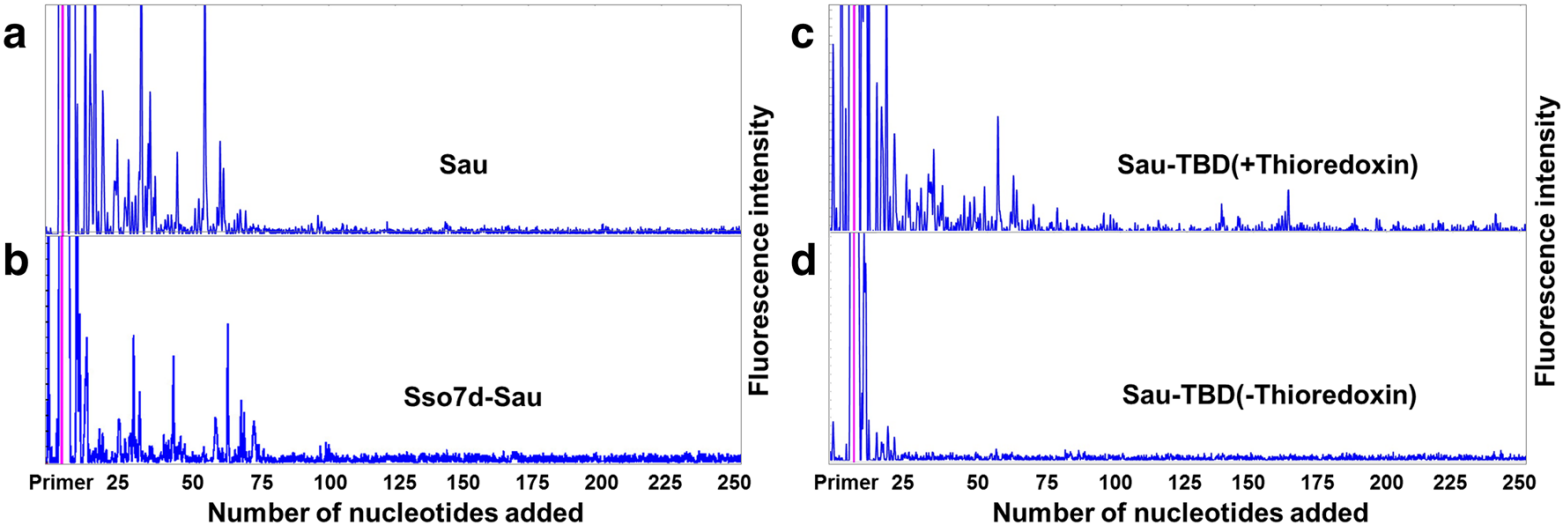
Electropherogram traces of Sau, Sso7d-Sau and Sau-TBD (−/+ thioredoxin) for processivity analysis. Each trace shows all of the amplified products in one lane, and each peak represents a mount of products of a particular length. The length of primer extension was determined according to the size maker that was run on the same gel and is labelled on the x-axis. **a** Electropherogram traces of Sau (20 pM). **b** Electropherogram traces of Sso7d-Sau (50 pM). **c** Electropherogram traces of Sau-TBD (50 pM) with thioredoxin (50 µM). **d** Electropherogram traces of Sau-TBD (50 pM) without thioredoxin

The processivities of Sau, Sso7d-Sau and Sau-TBD (−/+ thioredoxin) were quantified using the fluorescence-based sequencing assay described in the Sect. 2. Different ratios of polymerases to primed-templates were quantified at different time points until the medium length of the products became constant and the reactions reached the status of processive. In Fig. 2a–d, the electropherogram traces of Sau, Sso7d-Sau and Sau-TBD (−/+ thioredoxin) at processive conditions are shown, respectively. The majority of product extension lengths of Sau and Sso7d-Sau were shorter than 75 nt. In contrast, there were less products shorter than 75 nt, and significantly more products larger than 75 nt amplified by Sau-TBD with thioredoxin. Sau-TBD without thioredoxin could not amplify a significant amount of products larger than 25 nt.

The processivity parameter (PI) and the average primer extension length were calculated and are summarized in Table 2. The processivity parameter of Sau was 0.9726, which correlated to an average primer extension length of 36.4 nt. The processivity parameter of Sso7d-Sau was 0.9808, indicating an average primer extension length of 52.1 nt. The processivity of Sau-TBD with thioredoxin was 0.9856, indicating an average primer extension length of 74.2 nt. Intriguingly, the processivity of Sau-TBD without thioredoxin was 0.7135, which correlated to an average primer extension length of only 3.5 nt. Compared with the processivity parameter of Sau, it was shown that the insertion of TBD into Sau caused a one-tenth processivity decrease. While stimulated with thioredoxin, the processivity of Sau-TBD was dramatically enhanced to approximately twofold that of Sau.

### Comparison of the Amplifying Efficiencies of Sau, Sso7d-Sau and Sau-TBD (−/+ Thioredoxin)

**Fig. 3:**
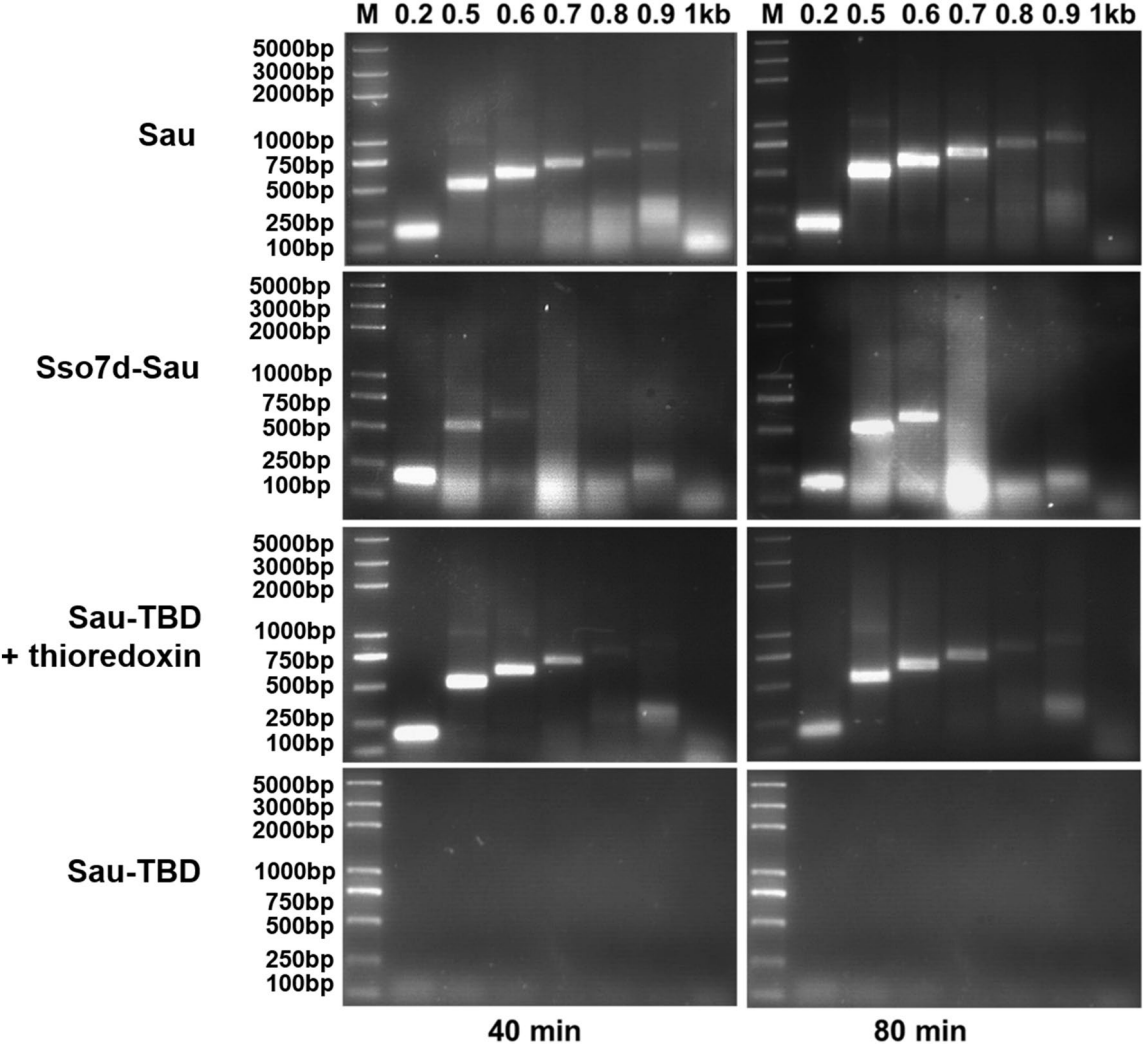
Comparison of the amplifying efficiencies of Sau, Sso7d-Sau and Sau-TBD (−/+ thioredoxin) in RPA. Lambda DNA (130 pg/µl), used as template, and pairs of oligonucleotides (Table 1) were used to amplify amplicons from 200 to 1000 bp (indicated at the bottom of the gel image). “M” indicates the molecular weight marker. Sau, Sso7d-Sau and Sau-TBD were used at a concentration of 0.44 µM, and thioredoxin was added to a concentration of 50 µM when needed. In the Sau reactions, the reagents were used according to the typical RPA reaction. In the Sso7d-TBD and Sau-TBD reactions, KAc was added to a final concentration of 140 mM, the other reagents remained the same as in the RPA reaction. The reactions were carried out at 37 °C for 40 min

To verify whether the enhanced processivity led to better amplifying efficiency in RPA, amplifying assays were carried out with lambda phage DNA as template and primer pairs to amplify amplicons from 200 to 1000 bp within a range of extension times. Equivalent molar amounts of DNA polymerases were used in comparing reactions. It is shown in Fig. 3 that Sau and Sau-TBD with thioredoxin failed to amplify amplicons larger than 900 bp, which indicated that the processive Sau-TBD did not necessarily exhibit better amplifying efficiency when stimulated with thioredoxin. In the absence of thioredoxin, Sau-TBD was not able to amplify amplicons as small as 200 bp, which confirmed the results of polymerase activity and processivity assays. Unexpectedly, Sso7d-Sau did not amplify any amplicons larger than 600 bp, even for extended amplification times.

### Comparison of the Salt Tolerances of Sau, Sso7d-Sau and Sau-TBD (−/+ Thioredoxin) in RPA

**Fig. 4:**
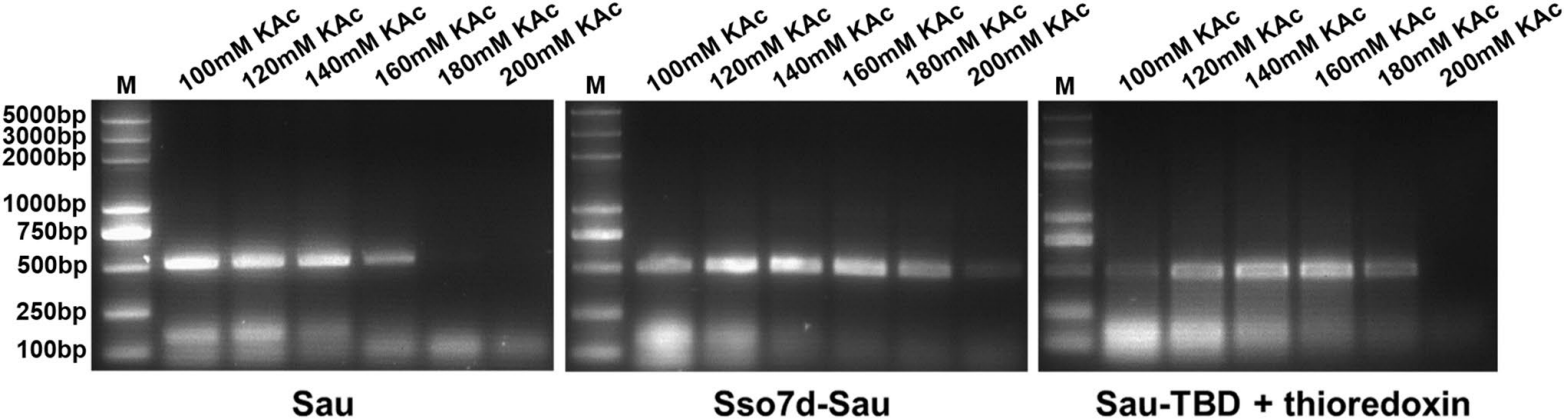
Comparison of the salt tolerance among Sau, Sso7d-Sau and Sau-TBD (+ thioredoxin) in the RPA reaction. Lambda DNA (130 pg/µl) was used as template and oligonucleotides 500_F and 500_R were used as primers. Sau, Sso7d-Sau and Sau-TBD were used at a concentration of 0.44 µM, and thioredoxin was added to a concentration of 50 µM when needed. Increasing concentrations of KAc were added into different reactions. “M” indicates the molecular weight marker. Apart from the concentration of KAc and the difference of the polymerase, other reagents were added following a typical RPA reaction. The reactions were carried out at 37 °C for 40 min

DNA samples are likely to contain inhibitors, such as salts, that significantly restrict the efficiency of amplification. There is a general thought that processive polymerases are more tolerant to high-salt buffer. To confirm the correlation between processivity and salt concentration, different amounts of KAc was added to RPA reactions, and the differences in amplifying efficiency between Sau, Sso7d-Sau and Sau-TBD with thioredoxin were compared (Fig. 4). As expected, the maximum concentration of KAc that Sau could bear was 160 mM, while Sso7d-Sau and Sau-TBD (+ thioredoxin) could amplify a significant amount of products at KAc concentrations of 200 and 180 mM, respectively. It was also revealed that both of two engineered Sau variants exhibited a shifted adaptive range of KAc concentration towards the high-salt direction when compared with the wild type Sau.

## Discussion

The fusion protein Sso7d-Sau displayed only 23% polymerase activity compared with the wild type Sau (Table 2), although it was proven by Wang et al. that the fusion of Sso7d to Taq did not significantly affect the catalytic activity [14]. Sso7d-Sau exhibited higher processivity than the wild type Sau; however, the extent of the processivity enhancement was not as significant as anticipated (Fig. 2a, b; Table 3). According to the results that were published by Wang Y. and colleagues in 2004, the fusion of Sso7d to full-length Taq led to a 3.7-fold enhancement of the average primer extension length, and the fusion of Sso7d to a truncated Taq (lacking the 5′→3′ exonuclease domain) led to a 16.6-fold enhancement of the average primer extension length [14]. We speculate that the working temperature of Sau was not optimum for Sso7d; therefore, the movement and DNA binding force of Sso7d were restricted at the lower temperature, causing the low polymerse activity of Sso7d-Sau. This speculation was verified by the amplifying efficiency assay. The size of the maximum amplicon of Sso7d-Sau was much smaller than that of Sau (Fig. 3), although the processivity of Sso7d-Sau was higher than that of Sau. The low activity of Sso7d that was caused by the non-optimum working temperature hindered the recruitment of Sso7d-Sau between cycles; therefore, the size of the maximum amplicon was reduced. The result is explainable, however non-predictable, since it was proven that the DNA binding accessary protein of T7 phage DNA polymerase works well at non-optimum temperature [11].

**Table 3:** Summary of the results of the processivity assay

Polymerase	Microscopic processivity (P_I_)	Average primer extension length (nt) [1/(1 − PI)]
Sau	0.9726 ± 0.0006	36.5 ± 0.8
Sso7d-Sau	0.9808 ± 0.001	52.1 ± 2.8
Sau-TBD(+ thioredoxin)	0.9865 ± 0.0001	74.1 ± 0.6
Sau-TRD(− thioredoxin)	0.7135 ± 0.004	3.5 ± 0.06

In the meantime, the TBD of T7 DNA polymerase was inserted into Sau in the corresponding place to produce a hybrid polymerase, Sau-TBD. It was anticipated that, like the T7 phage DNA polymerase, Sau-TBD would exhibit high processivity when stimulated with the processivity factor thioredoxin from *E. coli*. The insertion of TBD into Sau caused approximately 57.4% polymerase activity loss (Table 2), and the average primer extension length decreased from 36.4 to 3.5 (Table 3). We propose the dramatic decrease in polymerase activity and processivity was due to the interference with DNA binding by the added TBD. However, when stimulated by thioredoxin, the average primer extension length of Sau-TBD increased directly from 3.5 to 74.1 (Table 3), and the polymerase activity increased to a similar level to that of the wild type Sau (Table 2). The results coincided with the fact that, unlike Sso7d, which bound directly to dsDNA with a *K*d of 1–2 μM, thioredoxin prevented the polymerase from dissociating in a structure-forming manner [23, 24]. After forming a 1:1 complex with Sau-TBD, thioredoxin could easily slide with the DNA polymerase on the template; therefore, the nucleotide-incorporating catalysis was not hindered. Our results showed that the maximum amplicon size of Sau was approximately 900 bp and Sau-TBD could not amplify amplicons as small as 200 bp. Unexpectedly, we failed to amplify any amplicons larger than 900 bp using the Sau-TBD:thioredoxin complex, even for an extended amplification time. A similar result for an engineered Taq DNA polymerase was published by Davidson in 2003, who assumed that the maximum amplicon size was not limited by the processivity of the polymerase, but that the mismatches formed at the 3′ termini [11]. Nonetheless, the results we obtained could not be explained by this speculation, as the large fragment of Sau that we used encompassed the 3′→5′ exonuclease domain. In the paper published by Wang et al. in 2004, it was shown that fusion of the Sso7d protein to the mutant Taq lacking the 5′→3′ exonuclease domain or Pfu could significantly enhance their processivity and PCR performance. Fusing the Sso7d protein to the wild type Taq generated the most processive polymerases among all that were tested in that paper, although no significant PCR performance improvement was observed with Sso7d-Taq when compared with Taq [14]. Based on their findings and the results that we obtained, it is suggested that the attachment of a DNA binding accessory protein to less processive polymerases (Sau-TBD, Sso7d-TaqΔ289 and Pfu) was efficient in enhancing the amplification performance. However, enhancing the processivity of comparable highly processive polymerases (Sau and Taq) would not necessarily enhance the amplification performance. We believe that the polymerization efficiency is determined by two competing factors: the increase in processivity and the decrease in the dissociation rate of the polymerase from the replicated template. A reasonable level of processivity provides the polymerase enough strength to “grasp” onto the DNA template and efficiently perform the polymerization task and, therefore, is essential to amplify long amplicons within a limited time. Nevertheless, polymerases with excessively high processivity are difficult to be recycled and, therefore, are not able to amplify long amplicons as well.

Different polymerases function optimally under specific buffer conditions. There is a general thought that processive polymerases are more tolerant to high-salt conditions than less processive polymerases. Our results demonstrated that Sso7d-Sau and Sau-TBD (+ thioredoxin) exhibited higher salt tolerance than the wild type Sau. The upper limits of KAc concentration for Sau, Sso7d-Sau and Sau-TBD (+ thioredoxin) were 160, 200 and 180 mM, respectively (Fig. 4). For Sau, the optimum concentration of KAc was 100 mM, while Sso7d-Sau and Sau-TBD (+ thioredoxin) amplified limited amounts of product at 100 mM KAc and maximum amounts of product at approximately 140 mM KAc. This suggested that the adaptive range of KAc concentrations shifted towards the high-salt direction for Sso7d-Sau and Sau-TBD (+ thioredoxin). It was interesting to find that, although the processivity of Sau-TBD (+ thioredoxin) was much higher than that of Sso7d-Sau, the salt tolerance ability of Sau-TBD (+ thioredoxin) was less than that of Sso7d-Sau. We speculate the different mechanisms of processivity enhancement led to the differences in the enhancement of the salt tolerance ability between Sso7d-Sau and Sau-TBD (+ thioredoxin). Thioredoxin prevents the polymerase from dissociating in a structure-forming manner; therefore, it is more susceptible to the ionic strength provided by the buffer than the direct-DNA-binding factor Sso7d.

In conclusion, the present study demonstrated two methods for enhancing the processivity and salt tolerance of Sau, which is the polymerase optimized in the isothermal nucleic amplifying technique, RPA. However, both engineered polymerases, Sso7d-Sau and Sau-TBD (+ thioredoxin), failed to exhibit improved amplifying efficiency. Based on our findings and the results Yang et al. presented, it is indicated that it is critical to optimize the processivity of a specific DNA polymerase to improve its polymerization ability.

## Electronic supplementary material

Below is the link to the electronic supplementary material.

Comparison of amplifying efficiency when different amount of thioredoxin was added into typical RPA reactions with Sau-TBD as polymerase. Lambda DNA (130 pg/µl) was used as template and oligonucleotides 500_F and 500_R were used as primers. Sau-TBD was added instead of Sau in a typical RPA reaction at a concentration of 0.44 µM, the concentration of thioredoxin added into each reaction was indicated at the bottom of the picture. M indicates molecular weight marker. The reactions were carried out at 37 °C for 40 min. Supplementary material 1 (TIF 425 KB)

## Abbreviations

RPA: Recombinase polymerase amplification
Sau: *Staphylococcus aureus* Pol I
HSV: Herpes simplex virus
Bsu: *Bacillus subtilis* Pol I
TBD: Thioredoxin-binding domain
Bst: *Bst* DNA polymerase large fragment
