# Supplementary figures and images for: Two Approaches to Enhance the Processivity and Salt Tolerance of Staphylococcus aureus DNA Polymerase
Source: Protein J. 2019 Feb 13;38(2):190–8. doi: 10.1007/s10930-019-09818-7 (PMC6486907; doi:10.1007/s10930-019-09818-7)

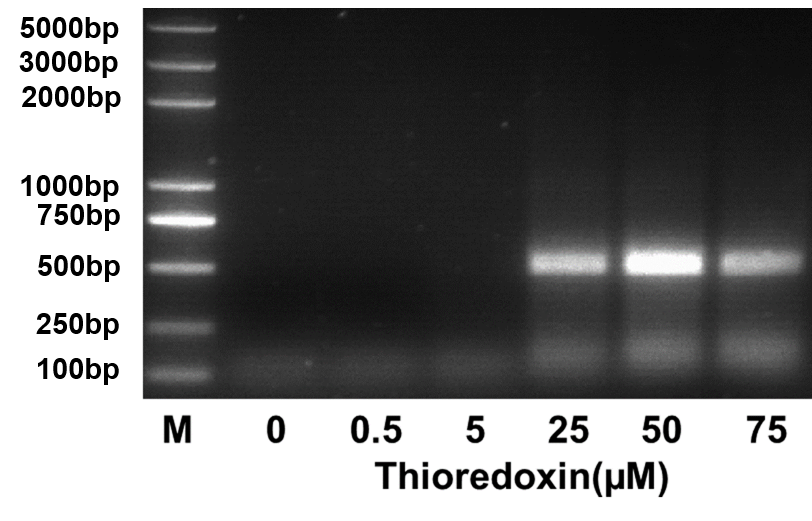

Supplement: Supplementary file 1 — Comparison of amplifying efficiency when different amount of thioredoxin was added into typical RPA reactions with Sau-TBD as polymerase. Lambda DNA (130 pg/µl) was used as template and oligonucleotides 500_F and 500_R were used as primers. Sau-TBD was added instead of Sau in a typical RPA reaction at a concentration of 0.44 µM, the concentration of thioredoxin added into each reaction was indicated at the bottom of the picture. M indicates molecular weight marker. The reactions were carried out at 37 °C for 40 min. Supplementary material 1 (TIF 425 KB) [file 10930_2019_9818_MOESM1_ESM.tif]
